# *Panx3* links body mass index and tumorigenesis in a genetically heterogeneous mouse model of carcinogen-induced cancer

**DOI:** 10.1186/s13073-016-0334-8

**Published:** 2016-08-09

**Authors:** Kyle D. Halliwill, David A. Quigley, Hio Chung Kang, Reyno Del Rosario, David Ginzinger, Allan Balmain

**Affiliations:** 1 Helen Diller Comprehensive Cancer Center, University of California, San Francisco, CA USA; 2 Department of Bioengineering and Therapeutic Sciences, University of California, San Francisco, CA USA; 3 Department of Epidemiology and Biostatistics, University of California, San Francisco, CA USA; 4 Invitae Corporation, 458 Brannan St, San Francisco, CA 94107 USA; 5 Thermo Fisher Scientific, 5791 Van Allen Way, Carlsbad, CA 92008 USA; 6 Department of Biochemistry and Biophysics, University of California, San Francisco, CA USA

**Keywords:** Carcinogenesis, BMI, Linkage analysis, Tumor susceptibility

## Abstract

**Background:**

Body mass index (BMI) has been implicated as a primary factor influencing cancer development. However, understanding the relationship between these two complex traits has been confounded by both environmental and genetic heterogeneity.

**Methods:**

In order to gain insight into the genetic factors linking BMI and cancer, we performed chemical carcinogenesis on a genetically heterogeneous cohort of interspecific backcross mice ((Mus Spretus × FVB/N) F1 × FVB/N). Using this cohort, we performed quantitative trait loci (QTL) analysis to identify regions linked to BMI. We then performed an integrated analysis incorporating gene expression, sequence comparison between strains, and gene expression network analysis to identify candidate genes influencing both tumor development and BMI.

**Results:**

Analysis of QTL linked to tumorigenesis and BMI identified several loci associated with both phenotypes. Exploring these loci in greater detail revealed a novel relationship between the Pannexin 3 gene (*Panx3*) and both BMI and tumorigenesis. *Panx3* is positively associated with BMI and is strongly tied to a lipid metabolism gene expression network. Pre-treatment *Panx3* gene expression levels in normal skin are associated with tumor susceptibility and inhibition of Panx function strongly influences inflammation.

**Conclusions:**

These studies have identified several genetic loci that influence both BMI and carcinogenesis and implicate *Panx3* as a candidate gene that links these phenotypes through its effects on inflammation and lipid metabolism.

**Electronic supplementary material:**

The online version of this article (doi:10.1186/s13073-016-0334-8) contains supplementary material, which is available to authorized users.

## Background

Elevated body mass index (BMI) has recently been highlighted as playing a profound role in the development of a diverse array of cancers. For some cancers, such as esophageal, endometrial, and uterine, BMI has been demonstrated to be associated with up to 40 % of all cases [1, 2]. In the USA, it is estimated that elevated BMI and obesity led to the development of 84,500 avoidable cancer cases and as many as 90,000 cancer deaths annually [1, 3].

A variety of hypotheses have been put forth to explain the increase in cancer risk associated with elevated BMI, including systemic inflammation concomitant with obesity, altered growth factor signaling and production, and altered sex hormone signaling and production [4–6]. The specific mechanism is likely to be dependent on the cancer type and it is probable that multiple factors cooperate to enhance cancer risk. Unfortunately, examining in detail the relationship between these two complex traits has been confounded by a combination of genetic complexity and the diverse environmental influences that must be taken into account. In order to address this paucity of data, we have analyzed the relationship between BMI and cancer incidence in a genetically heterogeneous mouse population. By crossing tumor susceptible *Mus musculus* (FVB) mice with tumor resistant *Mus spretus* (SPRET) mice we developed a genetically heterogeneous cohort of F1 backcross mice that varies significantly in both tumor susceptibility and BMI. Critically, this cohort was generated without using engineered susceptibilities to either obesity or tumorigenesis. This allows us to model more accurately the human condition of genetic heterogeneity and environmental (rather than genetic) tumor induction.

Using this cohort, we induced skin tumors using the chemical carcinogen 7,12-Dimethylbenz(a)anthracene (DMBA) and the tumor promoting agent 12-O-Tetradecanoylphorbol-13-acetate (TPA) [7]. This protocol initially results in the formation of benign papillomas, a fraction of which will progress into carcinomas. Previous studies have demonstrated that the tumors produced by this protocol are profoundly influenced by inherited susceptibility loci [8–12].

Comparing BMI and tumor burden in this cohort revealed a significant positive association in male mice. Using a systems approach involving QTL mapping, gene expression analysis, and gene expression correlation network analysis, we have been able to identify several candidate regions associated with both BMI and cancer. Further analysis of these regions identified Pannexin 3 (*Panx3*) as a strong candidate gene for the BMI/tumor susceptibility locus on chromosome 9. *Panx3* expression levels in normal skin are strongly associated with BMI and are also correlated with a number of genes within a well-conserved lipid metabolism network that may underlie effects on BMI. *Panx3* expression is also associated with tumor development and we demonstrate that inhibition of Pannexin function causes alterations in TPA-induced inflammatory signaling, providing a plausible mechanism for the relationship between *Panx3* and tumorigenesis.

## Methods

### Mouse breeding and husbandry

Male *Mus spretus* (SPRET/Ei) and female *Mus musculus* (FVB/N) mice obtained from the Jackson Laboratory were crossed to generate F1 hybrids. Female F1 mice were then crossed to male FVB/N mice to generate backcrossed mice, referred to as FVBBX mice. For mice undergoing tumor induction, tail tips were taken at seven weeks and snap frozen in liquid nitrogen for RNA and DNA. For mice not undergoing tumor induction, tail, dorsal skin, and other tissues were harvested after sacrifice by asphyxiation and cervical dislocation at seven weeks. All mouse experiments were approved by the University of California at San Francisco Laboratory Animal Resource Center.

### Tumor induction

Chemical carcinogenesis was initiated using the two-stage DMBA/TPA protocol. In brief, seven-week-old mice were shaved and treated with 25 μg DMBA in acetone over an approximately 2-in. squared region on the center of the back. For the following 20 weeks, 200 μL of 10^−4^ M TPA in acetone was applied twice per week to the DMBA-treated area. Mice were shaved two days prior to the first TPA treatment for that week. TPA treatments were continued for 20 weeks.

### Genotype, expression, and phenotype measurements

#### Phenotype measurements

Mice were weighed at seven weeks and values rounded to the nearest 100th of a gram. Lengths were measured by first anesthetizing mice and then by measuring the nasal–anal distance in centimeters rounded to the nearest tenth. BMI was calculated by taking the weight (in grams) at seven weeks and dividing by nasal to anal length squared (in cm).

The resulting phenotype values were mean centered by sex prior to use in analysis. All distributions were approximately normal. Litter size was not significantly correlated with any phenotype.

#### Tumor burden and carcinogenesis risk

Papillomas were counted every two weeks starting at the tenth week of TPA administration until the 20th week. This induction protocol also results in the development of carcinomas in a high fraction of mice. Mice were continually observed for the emergence of carcinomas. At carcinoma formation, the date and location were recorded. The time from DMBA/TPA treatment start to carcinoma emergence was then used to calculate carcinoma-free survival.

Tumor-bearing mice were sacrificed by asphyxiation followed by cervical dislocation when carcinoma diameter exceeded 1 cm and/or when the mouse body condition score began to deteriorate. At sacrifice, dorsal skin, all tumors, and other tissues were harvested. Tissues were immediately split, with one section fixed in 4 % paraformaldehyde and subsequently embedded in paraffin for histology, and another section snap frozen in liquid nitrogen for DNA and RNA extraction.

#### Genotyping

Genotypes were generated using pre-treatment tail DNA with a custom panel of TaqMan assays (Life Technologies, Carlsbad, CA, USA) consisting of 276 markers (Additional file 1: Table S1) tiling the autosomes and the X chromosome at approximately 10 cM spacing. Genotyping reactions were performed using the WaferGen SmartChip qPCR system (WaferGen Biosystems, Fremont, CA, USA) as per manufacturer’s recommendations. Autosomal genotypes were assigned by comparing sample values to control FVB, SPRET, and F1 samples.

For the X chromosome, genotypes were assigned by splitting mice by sex, manually identifying outliers, and defining genotype groups by linear discriminant analysis using the lda function from the *MASS* (7.3-33) package in R [13].

Prior to analysis, mice with high missing genotype rates, implausible recombination rates, and duplicate samples were identified and excluded. Additionally, markers with either low heterozygous or homozygous calls were excluded, as well as markers with high missing rates, as described in Broman and Sen [14].

#### Gene expression

RNA was generated from tail and dorsal skin samples taken at seven weeks. Tail tissue was frozen in liquid nitrogen, ground by chilled mortar and pestle, and suspended in TRIzol. TRIzol RNA extraction was then performed, followed by purification by Qiagen kit. RIN values were calculated by Bioanalyzer and samples with high quality (greater than six) were used for analysis.

Selected samples were hybridized to an Affymetrix mouse gene ST 1.1 array. Gene expression values were calculated in R using the *oligo* package (1.26.6) [15]. Expression values were normalized at the transcript level via the RMA algorithm using a set of custom set of probe annotations. This custom set of annotations was designed to account for the high quantity of sequence dissimilarities between FVB and SPRET mice by removing any probe with a known single nucleotide polymorphism (SNP) between these two strains from the annotation. This method has recently been published [16]. Expression levels were then normalized across plates via the COMBAT method [17].

### Bioinformatics analysis

#### QTL analysis

QTL analysis was performed using the scanone function from the R package *qtl* (version 1.27-10) [18]. Extended Haley-Knott regression was initially performed to identify single QTL for each phenotype in a single combined dataset consisting of both sexes. Significance was evaluated by comparing observed values to those obtained in 1000 random permutations for autosomes and 27,000 for the X chromosome.

Initial investigations compared the results of assessing each phenotype with no covariates, with sex as an additive covariate, and again as an interactive covariate. Including sex as an additive covariate was uninformative in all cases, owing to the phenotypes being mean-centered by sex. There was sufficient evidence to conclude that sex interacted meaningfully with genotype in several cases for BMI, and as a result sex was included as an interactive covariate for the autosomes for BMI. Sex was not included as a covariate for weight or length. LOD peak thresholds were defined by taking the 95 % Bayesian credible interval for each peak [14].

The existence of multiple loci and interactions between single loci was evaluated by using the scantwo function from *qtl* limited to chromosomes previously identified as having a significant QTL. The results were then compared against permutation thresholds to establish the existence of multiple loci in individual linkage peaks, as well as any *cis* or *trans* interactions between autosomal QTL. There was no evidence of any significant interactions between QTL, although some peaks were determined to be comprised of several independent signals.

In order to refine further the candidate QTL, we utilized the refineqtl function from the *qtl* package. This function attempts to maximize the LOD score by shifting the location of candidate loci in a combined model.

To assess the significance of sex:genotype interactions at individual QTL, we performed linear modeling incorporating marker genotype, sex, and the sex:genotype interaction term as predictors of phenotype values. Significance was evaluated by ANOVA with α = 0.05.

#### Candidate gene identification

Candidate genes associated with each QTL were identified by two strategies; first, by finding all genes located within the QTL peak that also possessed at least one non-synonymous SNP between FVB and SPRET animals; and second, by finding all genes with an expression QTL (eQTL) peak within the QTL region.

To identify genes with a SNP in a QTL, we used the markers closest to the QTL boundaries to define the physical boundaries of the QTL. Using these regions, we identified sites at which FVB and SPRET were non-equivalent in the GRCm38 (REL-1303) database of SNPs and indels available from the Sanger Institute (ftp://ftp-mouse.sanger.ac.uk/). We then annotated these sites with Annovar and excluded synonymous and non-coding sites [19]. The list of selected SNPs included any that resulted in one of the following: non-synonymous substitution, nonsense substitution, frameshift deletions, frameshift insertions, and variants in a splice site. Any gene with one or more of these types of polymorphisms was considered to be a candidate by sequence analysis.

To identify genes with significant *cis* or *trans* eQTL associated with the phenotype linked QTL, we performed differential expression analysis using the *siggenes* package (1.36.0) in R [20]. We calculated the d statistic for all genes relative to the marker closest to the QTL peak. For markers on the X chromosome we treated male and female genotypes as distinct (AA/AB for females, and AY/BY for males). Multiple test correction was applied by controlling the false discovery rate, with α approximately equal to 0.05.

Genes selected by differential expression analysis were then subjected to selection by eQTL peak analysis. For all genes that were significantly differentially expressed, we performed an eQTL analysis using the scanone function from the *qtl* package in R to identify the maximum LOD peak for that gene. If the Bayes 95 % credible interval for the eQTL overlapped with the 95 % credible interval for the associated phenotype QTL, and if the LOD score for the linkage peak was greater than or equal to three, the gene was considered to be a candidate by differential expression analysis.

#### Phenotype and gene expression analysis

We used the *limma* package (3.18.13) [21] to compare the expression levels of genes in the union of the two candidate gene sets (sequence and differential expression) to the respective phenotype values. We modeled gene expression values by phenotype values and adjusted for multiple tests by controlling the false discovery rate, with α approximately equal to 0.05. This was performed independently in males, females, and a combined dataset.

Genes with *p* values below the FDR were then adjusted for marker genotypes and compared to phenotype values. Genes that remained significant (α = 0.05) after adjusting for marker genotype were considered significant. For genes selected by eQTL analysis, we also evaluated the congruency of the QTL-phenotype/gene-eQTL/gene-phenotype relationships. In all cases except one, the QTL-phenotype/gene-eQTL/gene-phenotype was congruent with expectations.

Genes which were significant after accounting for marker genotype and which were congruent with expected directionality (if it was possible to evaluate this) were considered hits for each respective phenotype/sex combination.

#### Gene expression correlation network analysis

Gene expression values were correlated by Spearman’s method, and significance thresholds were estimated calculating the 5 % genome wide error rate (GWER) [22, 23]. Significance thresholds were calculated in male and female samples independently.

The *Panx3* neighborhood was defined as those probes correlated with *Panx3* with rho values over the 5 % GWER (first-degree neighbors), as well as all significant correlations of the set of first-degree neighbors.

Gene ontology enrichment analysis was accomplished by comparing genes in the *Panx3* neighborhood with all annotated probes using the R package *topGO* (2.14.0) [24].

#### Gene expression correlation network visualization

Gene co-expression networks were visualized in Cytoscape (3.2.1; http://www.cytoscape.org/) using default parameters and organic layout. Some node positions were then manually adjusted to improve interpretability.

### Panx inhibition and qPCR gene expression analysis

Forty female FVB mice aged seven weeks were broken into four treatment groups consisting of ten animals each. The mice were shaved across a two-in. square area on the back two days prior to treatment. The four groups received intraperitoneal (IP) injections of either 0.9 % saline or 100 mg/kg carbenoxolone (CBX; Sigma Aldrich C4790) dissolved in 0.9 % saline.

Thirty minutes after IP injections, mice were treated with either 200 μL of acetone or 200 μL of 10^−4^ M TPA in acetone across the shaved dorsal skin surface. At 24 h post topical treatment, mice were sacrificed and a section of dorsal skin snap frozen in liquid nitrogen and a section fixed in formalin for histology. RNA was then extracted using Direct-zol RNA MiniPreps (Zymogen; R2050).

Using this RNA, we then probed for the expression levels of *Il6* and *Il1b* (selected based on the observation that these genes were the most differentially expressed interleukins in a cohort of FVB mice treated with TPA) using TaqMan probes purchased from Life Technologies (Mm00446190_m1 for *Il6*; Mm00434228_m1 for *Il1b*). Beta-actin was included as a control probe.

Expression values were calculated by comparing the Ct values observed for *Il6* and *Il1b* to that of Beta-actin and compared to delta Ct values in the saline/acetone treated mice (via the delta-delta Ct method; http://www.bioconductor.org/packages/release/bioc/vignettes/ddCt/inst/doc/rtPCR.pdf). These values were then assessed for statistical significance by t test comparing the delta-delta Ct values between control and treatment groups. For plotting this was then converted into relative starting quantities by the following equation: 2^(−x), where x is the delta-delta Ct value.

Skin samples were fixed in 4 % PFA, dehydrated, and embedded in paraffin. Sections of 5 μm were mounted on slides and stained with hematoxylin and eosin (H&E). The degree of inflammation was evaluated by assessing the epidermal thickness and dermal nuclei abundance. Overall levels of inflammation were scored by a researcher blinded to sample labels on a scale of 0–3, with 0 corresponding to no inflammation and 3 to a high degree of inflammation. Differences between treatment groups were tested for significance by Fisher’s exact test.

### Statistical analysis

All statistical analyses were performed in R (3.0.2). Correlation analysis was performed using Spearman’s method. Differences between groups were evaluated by the Mann–Whitney U test. Linear regression significance was evaluated by ANOVA. The significance of phenotype-phenotype interactions was evaluated by incorporating both terms into a linear model and evaluating the significance of the interaction term.

Survival analysis was performed using the *survival* package (2.37-4) [25]. Cox proportional hazards modeling was performed on carcinoma-free survival data. Significance was evaluated by log-rank test.

## Results

### Increased weight and BMI are associated with increased papilloma burden

**Fig. 1:**
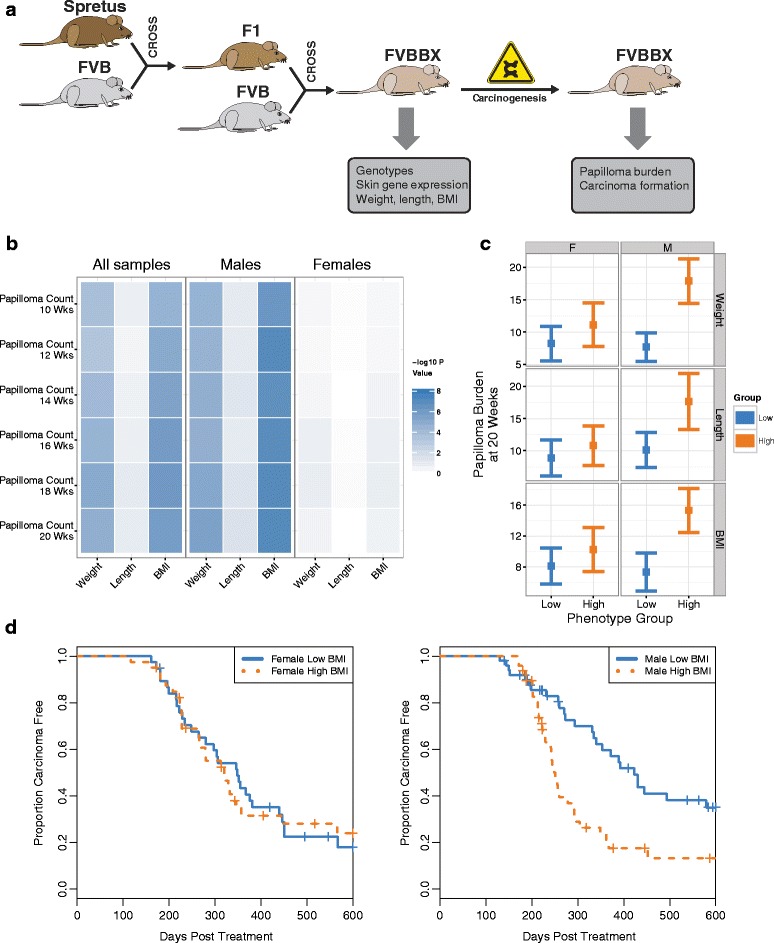
BMI and carcinogenesis are strongly associated in male mice. **a** *Schematic* of cross-generation and tumor induction. **b** Correlation *heatmap* between pre-treatment phenotype values and tumor burden measured at multiple tumor timepoints. Negative log10 of the p value for Spearman’s rank-order correlation is plotted for male mice, female mice, and the combined dataset. **c** Tumor burden is higher in male mice with higher BMI and weight values. Papilloma burden at 20 weeks was compared between mice in the highest and lowest quartile for each phenotype. *Bars* represent the 95 % CI for the mean for mice in the highest quartile (*blue*) and lowest quartile (*orange*) for each sex. Female and male mice are plotted separately on the *right and left halves*, respectively. All differences between phenotype groups in males are significant and no differences in females are significant. **d** *Survival curve* for mice with high BMI and low BMI values split by sex. *Kaplan–Meier plots* for carcinoma-free survival mice with the highest 25 % of BMI values (*orange*) and lowest 25 % (*blue*). BMI group is a significant predictor of carcinoma risk in male mice with *p* = 0.0013 by log-rank test. There is no significant difference in females

In order to evaluate the relationship between pre-treatment physiological parameters and carcinogenesis, we compared pre-treatment BMI, weight, and length to papilloma burden in two independent skin carcinogenesis cohorts. These cohorts were generated from a population of interspecific *Mus spretus* (SPRET/Ei) and *Mus musculus* (FVB/N) backcrossed mice. F1 progeny were generated by crossing male SPRET mice with female FVB animals, and the backcross population was generated by crossing female F1 mice with male FVB. The resulting population (FVBBX mice) was then phenotyped (see Methods) and subjected to two-stage chemical carcinogenesis (Fig. 1a).

The first cohort (hereafter Cohort 1) consisted of 141 mice, 139 of which had whole-genome genotyping data available. The second cohort of mice (Cohort 2) consisted of 237 mice, 235 of which had genotyping data available (Table 1). In both cohorts there were approximately as many female as male mice (Table 1). There were no significant differences in papilloma burden, BMI, or carcinoma risk between the two datasets. Additionally, sex was not significantly associated with papilloma burden or the risk of developing a carcinoma.

**Table 1:** Mouse totals by cohort and sex. Values in parenthesis represent the standard deviation

	Total mice	Mean weight (g)	Mean length (mm)	Mean BMI (g/cm2)	Mice with carcinomas	Mice with genotype data	Mice with expression data
Cohort 1 total	141	23.79 (4.85)	9.66 (0.58)	0.25 (0.04)	90	139	109
Cohort 1 males	78	26.13 (4.56)	9.85 (0.57)	0.27 (0.03)	45	76	59
Cohort 1 females	63	20.88 (3.41)	9.43 (0.52)	0.23 (0.03)	45	63	50
Cohort 2 total	237	24.05 (5.57)	9.60 (0.56)	0.26 (0.04)	172	235	0
Cohort 2 males	116	26.53 (5.53)	9.82 (0.53)	0.27 (0.03)	87	114	0
Cohort 2 females	121	21.68 (4.48)	9.39 (0.51)	0.24 (0.04)	85	121	0

In these cohorts, BMI and weight, but not length, were significantly positively correlated with papilloma burden (Fig. 1b). In a combined analysis, BMI was positively correlated to papilloma burden (*p* < 1e-5, rho = 0.25). This relationship appears to be driven primarily by male mice, which show a correlation *p* value < 1 e-7 and a rho of 0.38 (Fig. 1b). BMI was not significantly correlated with papilloma burden in female mice (Additional file 2: Figure S1). Similar results were observed for weight, which showed a significant positive correlation with papilloma burden in male mice (rho = 0.33; *p* < 1e-5), and no correlation in female mice. Separating the two cohorts showed a consistent level of correlation between BMI and papilloma burden for male mice in both cohorts: *p* < 1e-3 and rho = 0.4 for Cohort 1 male mice and *p* < 1e-3 with rho = 0.34 for Cohort 2 male mice.

Next, we compared papilloma burden between mice with extreme BMI values, defined as mice with BMIs in the top or bottom quartile of values for each sex. Comparing papilloma burden between mice with high and low BMI uncovered a significant increase in burden for male mice with high BMI (*p* < 1e-4; in this cohort high was defined as BMI greater than 0.29 and low as BMI less than 0.24), and no difference in female mice (Fig. 1c). This increase was consistent between the two cohorts (*p* < 1e-4 and *p* = 0.015 for cohorts 1 and 2, respectively). Male mice with weights in the highest quartile of values also showed a similar increase in papilloma burden (*p* < 1e-4 and *p* < 0.001 for cohorts 1 and 2, respectively).

This may suggest that the effect of increased weight and BMI on papilloma burden is attenuated, if not absent altogether, in female mice. In support of this, a linear model predicting papilloma burden by either BMI or weight and sex demonstrated the existence of a significant interaction between the two terms (*p* = 0.019 for the interaction between BMI and sex; *p* = 0.027 for weight and sex).

### Increased BMI is associated with carcinoma development

In addition to the influence of BMI on papilloma burden, we also investigated the relationship between BMI and carcinoma formation. For this investigation both cohorts were merged and treated as a single dataset in order to maximize statistical power.

Using this set of 378 mice, of which 262 developed a carcinoma, we found that BMI was significantly associated with increased risk of carcinoma development in male mice (*p* < 1e-3), but not in female mice. Male mice with high BMIs (as defined as the top 25 % of BMI values) were at approximately 2.36-fold increased risk of developing a carcinoma relative to mice with the 25 % lowest (*p* = 0.0013; 95 % CI for risk ratio: 1.40–4.00; Fig. 1d). Weight values were also associated with carcinoma-free survival, albeit at an attenuated level. In male mice weight predicted carcinoma-free survival with a *p* value = 0.026. In female mice there was no significant association between weight and carcinoma-free survival. Length was not a significant predictor for either sex (*p* > 0.05).

Carcinoma risk is defined both by the formation of benign lesions, as well as the progression of benign lesions to malignancy. To assess whether the effect of BMI on carcinoma risk was due to progression or initiation, we modeled carcinoma risk by both BMI and papilloma burden. In this model we found that BMI was not a significant predictor (*p* > 0.05), suggesting that the primary effect of BMI on tumorigenesis is to increase the number of early stage tumors rather than to induce tumor progression.

### Multiple overlapping genomic loci influence weight and BMI

**Fig. 2:**
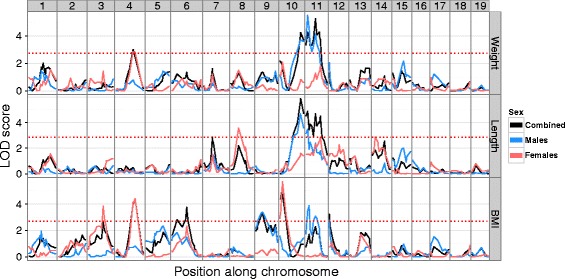
Genome-wide *linkage plots* for pre-treatment phenotypes by sex*.* Chromosomes are arranged head-to-tail on the *x-axis*, LOD scores are plotted on the *y-axis*. LOD scores were computed in all samples, as well as in males only (*blue*) and females only (*red*). Significance thresholds for the combined data are indicated by the *dashed horizontal red line*. The X chromosomes were omitted for this plot

FVB and SPRET mice vary significantly in average weight and adiposity, with FVB mice typically weighing more and having a lower percentage of body mass attributable to fat [26]. As these phenotypes are known to be genetically influenced, we next wanted to identify the genetic factors influencing BMI, weight, and length in the backcross population. To accomplish this, we performed QTL mapping using mice from both cohorts (see “Methods”). We were able to identify multiple significant loci for each phenotype (Fig. 2). For BMI, eight total loci were identified (on chromosomes 3, 4, 6, 9, 10, 11, 12, and X). For both weight and length, four loci were identified (for weight: chromosomes 4, 10, 11, X; for length chromosomes 7, 10, 11, and X). The effect of the most significant autosomal QTL for weight, length, and BMI is depicted in Additional file 2: Figure S2.

**Fig. 3:**
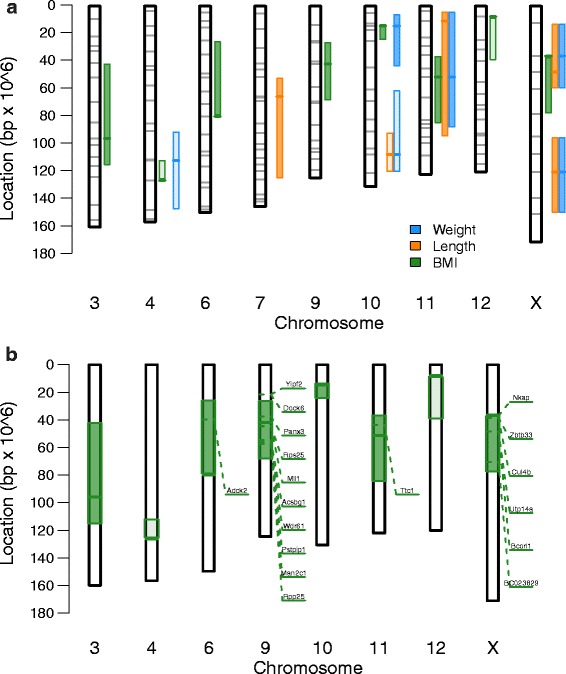
QTL for pre-treatment phenotypes and candidate BMI genes. **a** QTL regions by phenotype. *Shaded bounds* depict the QTL regions identified as significant for each phenotype, with the *colored bar* displaying the QTL region maximum. Marker locations are indicated as *gray bars* across the chromosome. The level of shading indicates QTL directionality, with *lightly shaded regions* corresponding to QTL with heterozygous mice having lower mean phenotype values and *heavily shaded regions* corresponding to QTL with heterozygous mice having higher mean phenotype values. **b** BMI associated genes. Candidate genes that were significantly associated with BMI are plotted, along with their location on the chromosome. QTL regions are plotted in *green*, with the QTL peak as a *thick dark line*. Shading is assigned as in panel A

We were able to determine that the weight QTL on chromosome 10 was composed of two distinct and opposite in orientation QTL, one distal and one proximal (Additional file 2: Figure S3). For both the weight and length QTL on chromosome X, we determined that the QTL comprised two distinct, but congruent in orientation, QTL. The final locations, LOD scores, and r^2^ values for all significant peaks are listed in Table 2, and the QTL regions are depicted graphically in Fig. 3a. In the majority of cases (14 out of 19), the SPRET allele conferred increased phenotype values, with the FVB allele associated with reduced values (Fig. 3a).

**Table 2:** QTL locations and variance explained

QTL name	Chromosome	Position (mb)	Left bound (mb)	Right bound (mb)	LOD score	Variance explained
BMI_3	3	96	41	116	3.81	0.031
BMI_4	4	126	111	126	5.05	0.055
BMI_6	6	80	25	80	3.81	0.024
BMI_9	9	42	25	69	3.88	0.027
BMI_10	10	15	13	25	6	0.059
BMI_11	11	51	36	85	3.89	0.027
BMI_12	12	8	8	40	3.58	0.039
BMI_X	X	36	36	78	6.76	0.098
WEI_4	4	112	91	148	3.51	0.044
WEI_10_PROX	10	15	5	44	4.74	0.028
WEI_10_DIST	10	107	60	121	3.58	0.052
WEI_11	11	51	4	89	5.31	0.063
WEI_X_PROX	X	36	12	60	9.13	0.194
WEI_X_DIST	X	120	95	151	4.35	0.137
LEN_7	7	66	52	126	3.18	0.033
LEN_10	10	107	91	121	4.18	0.07
LEN_11	11	11	4	95	4.62	0.06
LEN_X_PROX	X	48	12	60	5.61	0.174
LEN_X_DIST	X	120	95	151	5.62	0.151

QTL names were defined by combining the phenotype, the chromosome, and if applicable the relative location on the chromosome

Using linear models structured to permit an interaction between sex and marker genotype, we were able to identify two QTL with significant interactions with sex. BMI_3 and BMI_9, on chromosomes 3 and 9, respectively, both showed significant interactions between sex and genotype (BMI_3 p interaction = 0.020; BMI_9 p interaction = 0.038). BMI_3 showed a striking increase in BMI for heterozygous female mice relative to homozygous females, but no difference in male mice (Additional file 2: Figure S4). Conversely, heterozygous males at BMI_9 had approximately 8 % higher BMI than their homozygous counterparts, while female mice showed no difference between heterozygous and homozygous mice (Additional file 2: Figure S4). Two other loci, BMI_4 and BMI_11, were also suggestive but non-significant (Additional file 2: Table S2).

### BMI QTL peaks are associated with tumor burden in males

To evaluate the relationship between BMI linked QTL and tumorigenesis we used QTL genotypes as predictors of papilloma burden. This analysis was performed in a combined dataset, as well as sex-specific datasets.

We found that loci that influence BMI tend to also influence papilloma burden in males. Of the set of eight BMI QTL, six were significantly associated with papilloma burden in males. Of these six, four were not significantly associated with papilloma burden in female mice. There were two BMI QTL that were significantly associated with papilloma burden in all datasets (Additional file 2: Table S3).

In order to evaluate whether or not the relationship between these markers was congruent with the expectation of increased BMI positively influencing carcinogenesis risk, we assessed whether the high-BMI allele was also the high-papilloma burden allele. This was true in all cases except for BMI_3. The strongest and most consistent relationship between BMI QTL genotype and papilloma burden in both males and females was found at BMI_12 in a region containing a previously mapped papilloma susceptibility locus (*Skts5*) [10]. Of the remaining QTL, four out of six (BMI_4, BMI_6, BMI_9, and BMI_11) were associated with significant differences in papilloma burden by marker genotype in male mice. None of these QTL were significantly associated with papilloma burden in female mice.

### BMI_3 overlaps a papilloma QTL and confounds the relationship between BMI and tumorigenesis in female mice

In an effort to understand what differentiates the relationship between BMI and carcinogenesis in male and female mice, we further explored the female-specific QTL on chromosome 3 (BMI_3). This QTL, which extends from 21 cM/41 mb to 59 cM/116 mb, overlaps a previously identified tumor susceptibility QTL [8, 12]. Congruent with this observation, heterozygosity at this locus was associated with a significant decrease in papilloma burden in both males and females (*p* = 0.0012 in females and *p* = 0.0014 for males).

Conversely, while the effect of this QTL on papilloma burden was not different between the sexes, the effect on BMI was only present in females (*p* < 2e-5 in females, *p* = 0.44 in males). Importantly, heterozygous females were heavier than their homozygous counterparts. The combined effect of the BMI_3 QTL (increasing BMI in heterozygous mice) and the papilloma susceptibility QTL on chromosome 3 (decreasing papilloma burden in heterozygous mice) severely confounds the relationship between BMI and papilloma burden for females in this dataset.

After taking genotype at this QTL into account, there is evidence for a modest but significant positive correlation between BMI and papilloma burden for female mice (*p* < 0.01; rho = 0.19). There was no significant association with carcinoma risk after adjusting for BMI_3 marker genotype, suggesting that while there is a relationship between BMI and tumorigenesis in females in this dataset, it is attenuated relative to males.

In summary, we found that the relationship between papilloma burden and BMI is confounded in female mice by the existence of a female-specific BMI QTL that overlaps a previously described papilloma QTL. The result of this confounding is to mask the existence of a modest positive association between BMI and papilloma burden in female mice.

### Network analysis implicates Panx3 as a candidate lipid metabolism gene

QTL analysis can identify genomic regions linked to a phenotype, but this approach is limited by the low genetic resolution inherent in mouse backcross or intercross studies. Gene expression analysis can contribute substantially to identification of candidate genes or pathways that influence individual mouse phenotypes [22, 27–30]. In an attempt to identify the genetic elements responsible for the observed linkage to BMI, we performed gene expression correlation network analysis using a total of 110 pre-treatment tail skin samples from FVBBX mice. We selected genes inside each QTL region, in addition to those genes with significant eQTL linked to the BMI QTL. Using these genes as a starting point, we then identified genes with pre-treatment expression values significantly correlated with BMI (see “Methods”).

Analyzing gene expression values in this way allowed us to nominate several candidate genes associated with BMI (Fig. 3b; Additional file 2: Table S4). This set of genes includes the X-linked *Cul4b*, which has previously been associated with syndromic obesity in humans [31], and *Man2c1* which has been associated with a gonadal fat pad QTL on chromosome 9 in mice [32]. The set as a whole, however, was not significantly enriched for any specific function as assessed by gene ontology analysis.

As a strategy to gain additional insight into potentially novel associations between these candidate genes and adiposity, we performed gene expression correlation network analysis in pre-treatment mouse tail RNA. Briefly, each BMI-associated gene was used as a seed to propagate a gene co-expression network. The co-expression network consisted of the seed gene, genes significantly correlated to the seed gene (first degree neighbor genes), and genes significantly correlated to first degree neighbors. Co-expression networks were then analyzed for enrichment by gene ontology analysis (see “Methods”).

**Fig. 4:**
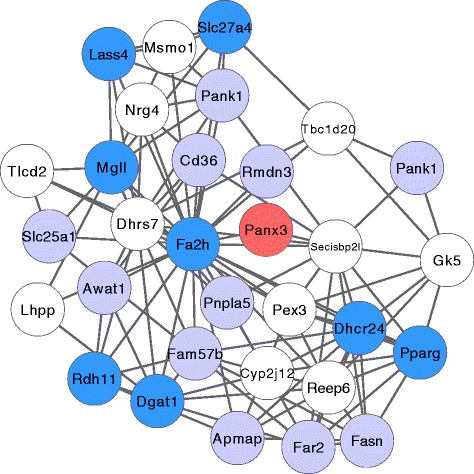
The *Panx3* gene expression correlation neighborhood. The *Panx3* gene expression correlation neighborhood is *plotted*. Genes are represented as *nodes* and significant correlations are plotted as *edges*. Nodes with GO annotations to lipid metabolic process (GO:0006629) are shown in *dark blue* and genes with other connections to adiposity are shown in *light blue. Panx3* is plotted in *red*. All other genes are plotted in *white*

The results of this analysis revealed a strong association between the *Panx3* gene and lipid metabolism genes. While other candidate genes have plausible relationships to BMI, none were members of clearly defined BMI-related gene co-expression networks. This led us to pursue *Panx3* as our primary candidate for further analysis. *Panx3* is a recently characterized integral membrane protein in the Pannexin family. This gene has previously described roles in keratinocyte differentiation and carcinogenesis, bone morphogenesis, WNT signaling, and monocyte recruitment [33–36]. *Panx3* expression was significantly correlated with BMI in males (*p* < 0.01; rho = 0.34) and not correlated in female mice (*p* > 0.05). Male mice in the upper quartile of BMI values mice had approximately 50 % higher expression than the lowest quartile (8.36 versus 7.99; *p* < 0.01). In addition, the gene expression correlation network surrounding *Panx3* was significantly enriched in genes associated with a variety of lipid phenotypes, including lipid metabolic process (GO:0006629; *p* < 1e-4), long-chain fatty acid transport (GO:0015909; *p* < 1e-4), and lipid storage (GO:0019915; *p* < 1e-3; Fig. 4). This enrichment was driven by numerous genes with established lipid metabolism annotations, including *Pparg*, *Fa2h*, *Mgll*, and *Dgat1*.

To assess the reproducibility of the *Panx3* correlation network, we analyzed gene expression data obtained from an independently derived cohort of FVBBX mice. This dataset consisted of RNA microarray data from 49 dorsal and 48 tail skins isolated from male mice taken between six and eight weeks. Using these data, we were able to confirm the reproducibility of the *Panx3* neighborhood to a strikingly high degree. In tail skins, the *Panx3* neighborhood in the validation cohort contained 31 out of 32 of the originally associated genes and in dorsal skins it contained 27 out of 32. In both cases the validation neighborhood was significantly enriched for the same type of lipid metabolism phenotypes as previously observed (lipid metabolic process GO:0006629; dorsal *p* < 1e-10, tail *p* < 1e-6: lipid storage GO:0019915; dorsal *p* < 1e-4, tail *p* < 1e-5). Although the association between *Panx3* and BMI was seen predominantly in male mice in this cohort, the linkage of *Panx3* expression to the lipid metabolism network was significant for both males and females (Additional file 2: Table S4).

Analyzing the sequence of the *Panx3* gene revealed the existence of three non-synonymous SNPs between the FVB and SPRET strains (Additional file 2: Table S6). These SNPs were not predicted to be deleterious to protein function (as assessed by PROVEAN; http://provean.jcvi.org), but are in close proximity to predicted phosphorylation sites [37]. In the case of the G500C/S167T polymorphism, the SNP occurs at the exact site of predicted phosphorylation.

### *Panx3* expression is positively associated with tumor risk

In order to evaluate the relationship between *Panx3* and tumorigenesis, we assessed whether pre-treatment levels of *Panx3* were associated with tumor development or progression. *Panx3* expression in pre-treatment tail skin was positively correlated with papilloma burden (*p* = 0.032, rho = 0.28; Additional file 2: Figure S5A), as well as with carcinoma risk in males (*p* = 0.012; Additional file 2: Figure S5B), indicating that elevated *Panx3* levels are associated with both early and late stages of tumor development. There was no significant relationship between *Panx3* expression and papilloma burden in female mice (*p* > 0.05, rho = −0.09; Additional file 2: Figure S5A) and *Panx3* expression was not a significant predictor of carcinoma risk for female mice (*p* > 0.05; Additional file 2: Figure S5C).

**Fig. 5:**
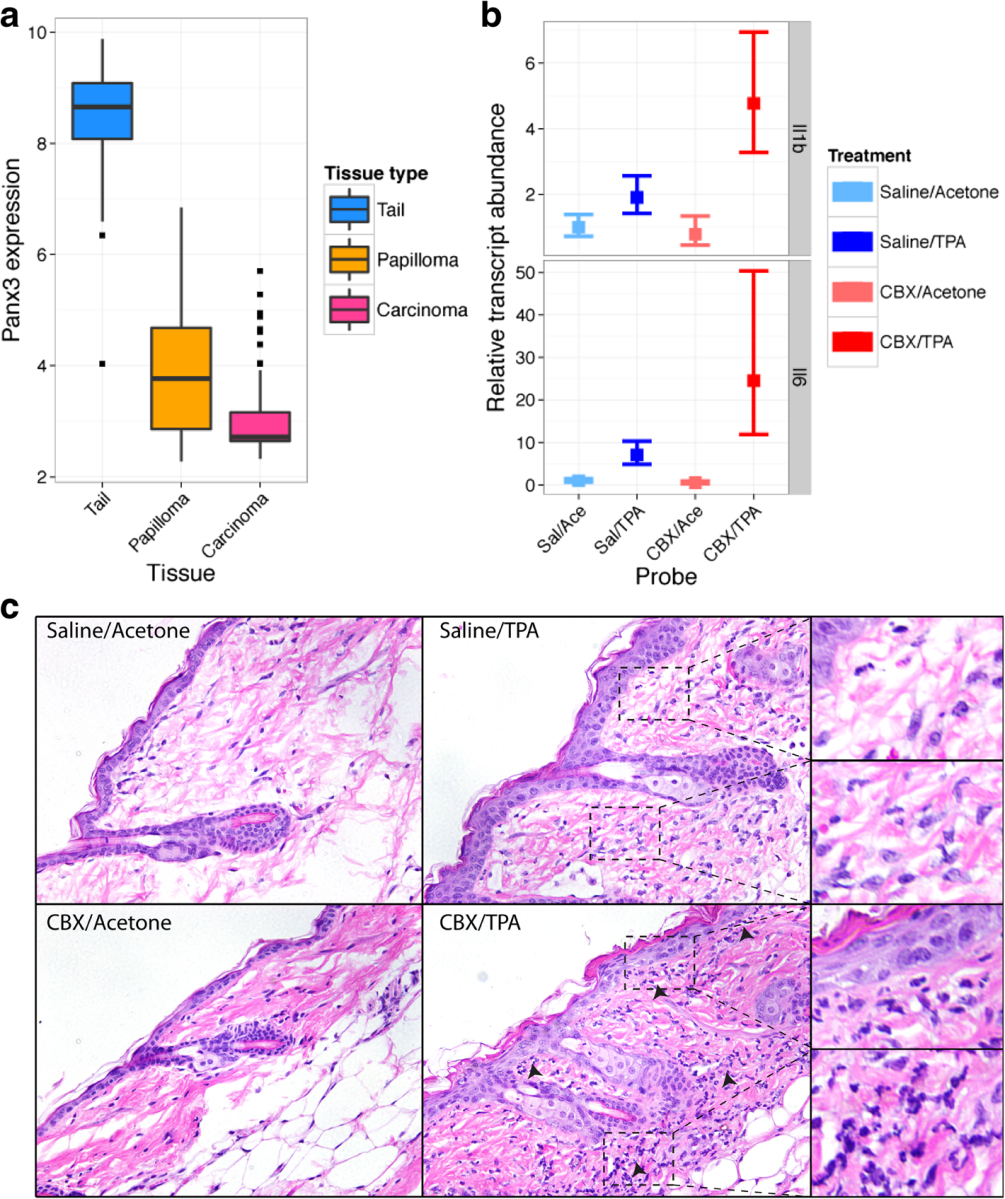
*Panx3* expression during tumorigenesis and the effect of Panx inhibitors on inflammatory interleukin expression. **a** *Panx3* expression is plotted by tissue. Expression levels are depicted by *box-and-whiskers plots*, with values lying further than 1.5 times the interquartile range plotted as *dots*. Comparisons between all tissues are significant; Tail vs. Papillomas, *p* < 1e-10; Tail vs. Carcinomas *p* < 1e-10; Papillomas vs. Carcinomas, *p* < 3e-5. **b** Interleukin 6 (*Il6*) and interleukin 1b (*Il1b*) transcript abundances relative to control mice are plotted following TPA and/or carbenoxolone (CBX) treatment. Mice were treated with IP saline or 100 mg/kg CBX, followed by 200 μL topical acetone or TPA in acetone. Control mice were defined as mice treated with IP saline and topical acetone. Tissues were harvested 24 h after topical treatment with TPA or acetone. Expression levels are shown as ddCt values transformed to relative transcript abundances. *Bars* represent the 95 % CI around the mean, with the mean plotted as a *square*. **c** Tissue histology following TPA and/or CBX treatment. Representative skin samples from mice treated as described above are displayed for each treatment group. *Arrows* denote high areas of infiltration in representative samples from mice treated with the combination of TPA and CBX, as assessed by an observer blinded to sample IDs. Several areas showing representative levels of infiltrating cells are enlarged for the TPA/acetone and TPA/CBX treated samples

*Panx3* expression has previously been reported to undergo downregulation during carcinogenesis in human tumors [34]. Using a previously generated cohort of mouse tail skin, benign papillomas, and malignant carcinomas, we assessed the expression levels of *Panx3* [30]. Congruent with human data, *Panx3* expression was markedly reduced in tumors, with carcinomas showing no significant expression of *Panx3* (Fig. 5a; *p* < 1e-10). Expression levels in papillomas were reduced, but less consistently than carcinomas (Fig. 5a; *p* < 3e-5). The positive association between tumor risk and *Panx3* levels in normal skin, but reduced *Panx3* expression in papillomas and carcinomas, supports the interpretation that the primary role of *Panx3* during tumor development is in influencing tumor susceptibility rather than tumor maintenance.

### Panx inhibition increases inflammation following TPA treatment

Among the functions attributed to members of the Pannexin family, particularly *Panx1* and *Panx3*, are roles in innate immune cell recruitment and inflammation [38–40]. Since inflammation is also known to play a critical, if complex, role in skin tumor development, we speculated that inhibition of Pannexin function may perturb inflammatory processes and thus influence development of skin tumors.

In order to test this hypothesis, we assessed the effect of small molecule inhibitors of Panx function (both *Panx1* and *Panx3*) on inflammatory cytokine expression following TPA treatment of mouse skin. To accomplish this, we used the Panx inhibitor carbenoxolone (CBX), which acts as an inhibitor of Pannexin channels [41–43]. Mice were pre-treated by intraperitoneal injection of CBX prior to the induction of inflammation with TPA. In mice treated with both CBX and TPA, the expression levels of the inflammatory interleukins *Il6* and *Il1b* were dramatically elevated (Fig. 5b) compared to control animals treated with either CBX alone or TPA alone. While there was no change in either *Il6* or *Il1b* expression following CBX treatment alone (*p* > 0.05 for both markers) treatment with TPA, as expected, significantly elevated the expression levels of both markers (*p* < 1e-5 for *Il6*; *p* = 0.0232 for *Il1b*), but not to the same extent as the combined treatment (*p* = 0.0232 for *Il6*; *p* = 0.00288 for *Il1b*). In agreement with these results, histological examination of the skin morphology in treated samples supported these claims, with CBX and TPA treated samples scoring significantly higher for markers of inflammation (infiltration into the upper dermis and epidermis) than TPA alone (*p* = 0.005; Fig. 5c). There was no difference between saline and CBX treated samples in the absence of TPA (*p* > 0.05). These results demonstrated that inhibition of Pannexin function can exacerbate the innate immune response to treatment with the tumor promoter TPA and could explain the observed genetic linkage between *Panx3* levels and tumor burden.

## Discussion

### Genetic relationships between BMI and cancer

The relationship between obesity and cancer has been a topic of intense scrutiny in the scientific community for well over a decade. In the intervening period since the first large-scale prospective investigation of this association and today, a myriad hypotheses have been proposed to account for this strong positive correlation [4–6]. However, due to the complexities intrinsic to both phenotypes, directly testing these hypotheses has proven challenging.

In this work we have employed a mouse model that is dependent on exposure to exogenous carcinogens and tumor promoters to mimic the environmental influence on cancer development. In addition, our protocol involves crosses between highly divergent mouse species to replicate the genetic heterogeneity in human populations [44]. The combination of these two factors (genetic heterogeneity and carcinogen induction in the absence of engineered tumor susceptibility) offered us a unique opportunity to study the interplay between BMI and cancer development in a system that more closely recapitulates the human condition.

Our results have confirmed the location of several previously described BMI and adiposity QTL, as well as described the existence of two novel loci. Our loci on chromosomes 3, 4, 9, 10, 11, and X appear to overlap previously described loci [45–56]. In the case of the QTL on chromosome 6 and chromosome 12, there did not appear to be an overlapping previously described BMI-associated QTL, suggesting these sites may be novel.

### *Panx3* is in a lipid metabolism network associated with BMI

In this work we have also identified a potential role for *Panx3*, a member of the recently described Pannexin gene family, in both BMI and cancer susceptibility. This gene family consists of three members (*Panx1–3)* in both rodents and humans [57]*.* The Pannexin genes have been associated with a variety of functions, including keratinocyte differentiation, wound healing, carcinogenesis, apoptosis, monocyte recruitment, inflammation, and osteoblast differentiation [34–36, 58–60]. *Panx3* and *Panx1* are known to work in conjunction with one another, both by directly interacting at the protein level as well as by coordinated gene expression regulation [40, 59].

Currently, there are no published functional links between *Panx3* activity and lipid metabolism. However, several key observations support the existence of this relationship. First, previous work from this laboratory has tied *Panx3* expression to adipocyte signaling and adiposity [61]. In this study we used gene expression network analysis of normal skin from a separate population of F1 backcross mice to predict, and subsequently verify, a relationship between the protein kinase *Hipk2* and adipocyte signaling and differentiation [61]. The expression network identified in this work also contained *Panx3*, suggesting that this gene may also contribute to these phenotypes. Network analysis has previously been applied to the identification of candidate genes involved in BMI [62, 63], demonstrating that this technique is well-suited to exploring the relationship between *Panx3* and BMI.

Further evidence for a role for Pannexins in lipid metabolism came from recent analysis of a *Panx1* knockout mouse, which acquired significantly increased amounts of subcutaneous adipose tissue, accompanied by a dramatic elevation of *Panx3* expression. These data, together with the observation of increased *Panx3* expression in mice fed a high-fat diet [35] strongly support the hypothesis that *Panx3* signaling is associated with increased adiposity [59] and consequently BMI. Additional observations have implicated *Panx3* in the persistent disseminated inflammation and metabolic syndrome associated with obesity [35]. Finally, in humans the genomic region containing *PANX3* has been previously associated with the development of obesity, as well as diabetes and serum adiponectin concentrations [64–67].

Although it is possible that *Panx3* influences adiposity through direct expression in skin adipocytes, an alternative explanation is that *Panx3* is primarily expressed in keratinocytes, possibly facilitating the important role played by lipid synthesis in maintenance of skin barrier function [68]. Indeed, previous studies have detected *Panx3* expression in suprabasal keratinocytes and analysis of subcutaneous adipocytes from mouse skin showed that *Panx3* is expressed at very low levels in this tissue (data not shown).

Understanding how lipid metabolism in the skin influences total BMI may be informed by recent publications detailing the influence of skin-specific knockouts of stearoyl-Coenzyme A desaturase 1 (*Scd1*) on global metabolism [69]. Skin-specific ablation of *Scd1* has dramatic whole-body effects on energy expenditure and lipid metabolism, rendering these mice profoundly resistant to obesity induced by a high-fat diet. In addition, these mice show substantial increases in global energy expenditure. Interestingly, the expression profile of *Panx3* is similar to *Scd1*, with strong expression in the sebocytes and liver in adult mice. The gene expression levels of these two genes are also significantly correlated in normal FVBBX tail skins (*p* < 2e-6; rho = 0.45). It is unclear from the available data what mechanism links skin-specific expression of *Scd1* to whole-body energy expenditure. However, these findings raise the possibility that skin-specific *Panx3* expression, much like *Scd1* expression, may influence whole-body energy expenditure and metabolism.

### Pannexins, inflammation, and tumor susceptibility

*Panx3* has been shown to influence monocyte recruitment directly [35]. By modulating recruitment, it is possible that *Panx3* may influence TPA-induced inflammation. An additional possibility for how *Panx3* could be influencing tumor development is through its relationship with *Panx1. Panx1* is known to play a major role in inflammation [38], an essential component of tumor formation using the DMBA/TPA carcinogenesis model [70]. *Panx3* may therefore influence susceptibility to tumorigenesis through modulating the response to inflammation, either alone or in concert with *Panx1*. In support of a role for Pannexins in TPA-mediated inflammation in the skin, treatment with the Pannexin inhibitor CBX resulted in a dramatic increase in morphological indications of inflammation as well as elevation of *Il6* and *Il1b* expression levels. CBX is known to inhibit other targets, including Connexin proteins. However, TPA is also a potent inhibitor of Connexin proteins [71–73], suggesting that in this context the effect of CBX on inflammation was driven by inhibition of Pannexin proteins. These results demonstrate that Pannexin signaling normally attenuates acute inflammatory responses in the skin following TPA treatment.

This conclusion may appear to contradict prior work demonstrating a positive relationship between Pannexins and inflammation [35, 74–76], as well as inflammation and tumor susceptibility [77]. However, interpretation of these data is complicated by the diverse roles of acute and chronic inflammatory responses in promoting or inhibiting skin cancer development. Mice that had undergone selective breeding for high acute inflammatory responses in the skin were shown to be resistant to skin carcinogenesis [78]. Similarly, over-expression of interleukin 1 alpha (*Il1a*) in skin using a keratin 14*-Il1a* transgene conferred complete resistance to the two-stage DMBA/TPA protocol for tumor development by a mechanism that was proposed to involve increased neutrophil infiltration [79], the phenotype also induced in the present studies by Pannexin inhibition.

Our results demonstrating increased TPA-induced infiltration after CBX treatment contrast with those from alternative assays of inflammation, which showed an inhibitory effect of CBX [71–73]. Clearly, both tissue context and the mechanism by which inflammation was induced must be taken into account in interpreting these data. TPA is a strong tumor promoting agent known to disrupt Connexin/gap junction activity [80], which in turn may feed back to alter Pannexin signaling [38]. While the details of interactions between *Panx1*, *Panx3*, and inflammation in skin remain to be elucidated, these data provide support for the proposal that alterations in *Panx3* levels and/or signaling contribute to skin tumor susceptibility through effects on acute inflammatory responses.

### Effects of sex on BMI and tumor burden

In our study we have observed a profound dichotomy between the sexes. In male mice, elevated BMI is strongly and positively associated with increased tumor burden and carcinoma risk. However, in females, there was no such relationship. A significant complication of this finding is the relationship between BMI_3 and papilloma burden. This BMI QTL overlaps with a previously described papilloma QTL, which happens to have opposite orientation to the BMI QTL. This BMI QTL is also a female-specific QTL. The net effect of this is to heavily confound the relationship between BMI and tumor burden for female mice in this cohort. After accounting for this relationship, there appears to be an attenuated, but significant, relationship between BMI and carcinogenesis in female mice.

This intriguing finding mirrors the state in human cancer, where a wide variety of cancers show an increased influence of BMI on risk in males relative to females. In a recent study involving over five million patients in the UK, Bhaskaran et al. assessed the influence of a variety of covariates on cancer risk [2]. The authors found that for 17 of the 22 cancers studied, BMI played a significant role in the risk of development. When the authors considered risk independently between males and females, they found that for all situations in which there was a significant difference in the influence of BMI on cancer risk by sex, males were at elevated risk. These results suggest, in line with our own observations, that there may exist a global difference in the relationship between BMI and cancer by sex.

Future studies of this cohort of genetically heterogeneous mice employing a more extensive expression dataset may help in the identification of additional candidate genes that will aid our understanding of the complex interactions between sex, obesity, and tumorigenesis.

## Conclusions

We have performed a large-scale analysis of the relationship between BMI and tumorigenesis in a genetically heterogeneous cohort of mice. The results of this experiment have demonstrated that, as in humans, these two phenotypes are highly correlated. Genome-wide linkage analysis identified several regions associated with BMI in this cohort, several of which were also associated with tumor burden.

Further, using a combined approach involving linkage analysis, gene expression analysis, and gene co-expression network analysis, we have implicated the *Panx3* gene as simultaneously influencing both phenotypes. In the skin pre-treatment *Panx3* expression levels are correlated with both BMI and tumor burden. *Panx3* is also present in a well-conserved lipid metabolism network and Pannexin function attenuates inflammatory signaling. These results strongly suggest that *Panx3* links tumorigenesis and BMI at the genetic level. Overall, our data show that the relationship between BMI and tumorigenesis is highly complex and represents the product of interactions at both the environmental and genetic levels.

## Abbreviations

BMI, body mass index; CBX, carbenoxolone; DMBA, 7,12-Dimethylbenz(a)anthracene; eQTL, expression QTL; GWER, genome wide error rate; QTL, quantitative trait loci/locus; SNP, single nucleotide polymorphism; TPA, 12-O-Tetradecanoylphorbol-13-acetate

## Additional files

Additional file 1: Table S1. Genotype marker locations and associated rsIDs. An Excel file (.xlsx) containing information about the genotyping markers used in this analysis. (XLSX 66 kb) Additional file 2: Supplementary figures S1-S5 and supplementary tables S2-S6. Supplementary figures and supplementary tables S2-S6. A Word (.docx) document containing all supplementary figures and supplementary tables S2-S6. **Table S2:** Sex interactions by QTL. Difference refers to the difference in means between heterozygous and homozygous mice. **Table S3**: BMI QTL and papilloma burden. **Table S4**: Genes significantly associated with BMI. **Table S5**: *Panx3* network gene correlation levels by sex. **Table S6**: *Panx3* polymorphisms between Spret and FVB mice. **Figure S1**: Twenty-week papilloma burden by BMI for male and female mice. **Figure S2**: QTL effect for the strongest autosomal QTL for each phenotype by sex for raw and mean-centered phenotype values. **Figure S3**: Proximal and distal regions of chromosome 10 influence weight in opposing directions. **Figure S4**: Effect of sex-specific QTL on BMI. **Figure S5**: *Panx3* expression and tumor development. (DOCX 224 kb) Additional file 3: Table S7. Gene expression phenotypes and sample IDs. This Excel file (.xlsx) contains phenotype information for the cohort of mice used for expression analysis. The samples are labeled both by mouse ID as well as by.CEL name, corresponding to the.CEL files available from the GEO site under access number GSE52650. (XLSX 90 kb)
