# Supplementary material for: Panx3 links body mass index and tumorigenesis in a genetically heterogeneous mouse model of carcinogen-induced cancer
Source: Genome Med. 2016 Aug 9;8:83. doi: 10.1186/s13073-016-0334-8 (PMC4977876; doi:10.1186/s13073-016-0334-8)
Supplement: Additional file 2: Supplementary figures S1-S5 and supplementary tables S2-S6. — Supplementary figures and supplementary tables S2-S6. A Word (.docx) document containing all supplementary figures and supplementary tables S2-S6. Table S2: Sex interactions by QTL. Difference refers to the difference in means between heterozygous and homozygous mice. Table S3: BMI QTL and papilloma burden. Table S4: Genes significantly associated with BMI. Table S5: Panx3 network gene correlation levels by sex. Table S6: Panx3 polymorphisms between Spret and FVB mice. Figure S1: Twenty-week papilloma burden by BMI for male and female mice. Figure S2: QTL effect for the strongest autosomal QTL for each phenotype by sex for raw and mean-centered phenotype values. Figure S3: Proximal and distal regions of chromosome 10 influence weight in opposing directions. Figure S4: Effect of sex-specific QTL on BMI. Figure S5: Panx3 expression and tumor development. (DOCX 224 kb) [file 13073_2016_334_MOESM2_ESM.docx]

Title

***Panx3* links body mass index (BMI) and tumorigenesis in a genetically heterogeneous mouse model of carcinogen-induced cancer**

Kyle D. Halliwill, Helen Diller Comprehensive Cancer Center, University of California, San Francisco, San Francisco, California; Department of Bioengineering and Therapeutic Sciences, University of California San Francisco,

David Quigley, Helen Diller Comprehensive Cancer Center, University of California, San Francisco; Department of Epidemiology and Biostatistics, University of California, San Francisco

Hio Chung Kang, Invitae Corporation, 458 Brannan St, San Francisco, CA, 94107

Reyno Del Rosario, Helen Diller Comprehensive Cancer Center, University of California, San Francisco

David Ginzinger, Thermo Fisher Scientific, 5791 Van Allen Way, Carlsbad, CA 92008

Allan Balmain, Helen Diller Comprehensive Cancer Center, and Department of Biochemistry and Biophysics, University of California, San Francisco

Running Title

***Panx3* links BMI and tumorigenesis**

Supplementary tables

Additional file 2: Table S2: Sex interactions by QTL. Difference refers to the difference in means between heterozygous and homozygous mice.

| QTL Name | Chromosome | Male AA | Male AB | Male difference | Female AA | Female AB | Female difference | p value |
| --- | --- | --- | --- | --- | --- | --- | --- | --- |
| BMI_3 | 3 | 0.27 | 0.27 | 0 | 0.23 | 0.25 | -0.02 | 0.02 |
| BMI_4 | 4 | 0.28 | 0.27 | 0.01 | 0.25 | 0.23 | 0.02 | 0.088 |
| BMI_6 | 6 | 0.27 | 0.28 | -0.01 | 0.23 | 0.24 | -0.01 | 0.576 |
| BMI_9 | 9 | 0.26 | 0.28 | -0.02 | 0.24 | 0.24 | 0 | 0.038 |
| BMI_10 | 10 | 0.27 | 0.28 | -0.01 | 0.23 | 0.25 | -0.02 | 0.149 |
| BMI_11 | 11 | 0.26 | 0.28 | -0.02 | 0.24 | 0.24 | 0 | 0.065 |
| BMI_12 | 12 | 0.28 | 0.26 | 0.02 | 0.24 | 0.24 | 0.01 | 0.232 |
| BMI_X | X | 0.26 | 0.28 | -0.03 | 0.23 | 0.25 | -0.02 | 0.128 |

Additional file 2: Table S3: BMI QTL and papilloma burden

| QTL Name | Chromosome | Combined | Male only | Female only | Congruent |
| --- | --- | --- | --- | --- | --- |
| BMI_3 | 3 | Significant | Significant | Significant | FALSE |
| BMI_4 | 4 | Significant | Significant | NS | TRUE |
| BMI_6 | 6 | Significant | Significant | NS | TRUE |
| BMI_9 | 9 | NS | Significant | NS | TRUE |
| BMI_10 | 10 | NS | NS | NS | NA |
| BMI_11 | 11 | NS | Significant | NS | TRUE |
| BMI_12 | 12 | Significant | Significant | Significant | TRUE |
| BMI_X | X | NS | NS | NS | NA |

Additional file 2: Table S4: Genes significantly associated with BMI.

| Probe ID | Symbol | Chromosome | QTL source | Candidate source | Sex significance | Post adjustment p value |
| --- | --- | --- | --- | --- | --- | --- |
| 10385343 | Ttc1 | chr11 | BMI_11 | SNV | C | 6.00E-05 |
| 10385343 | Ttc1 | chr11 | BMI_11 | SNV | M | 0.00057 |
| 10537441 | Adck2 | chr6 | BMI_6 | SNV | C | 0.00105 |
| 10583676 | Yipf2 | chr9 | BMI_9 | eQTL | M | 0.00656 |
| 10591614 | Dock6 | chr9 | BMI_9 | eQTL | M | 0.00744 |
| 10592355 | Panx3 | chr9 | BMI_9 | SNV | M | 0.04289 |
| 10592856 | Rps25 | chr9 | BMI_9 | eQTL | M | 0.01158 |
| 10592942 | Mll1 | chr9 | BMI_9 | SNV | M | 0.01482 |
| 10593723 | Acsbg1 | chr9 | BMI_9 | BOTH | M | 0.03447 |
| 10593740 | Wdr61 | chr9 | BMI_9 | eQTL | M | 0.04393 |
| 10585555 | Pstpip1 | chr9 | BMI_9 | SNV | M | 0.03534 |
| 10585652 | Man2c1 | chr9 | BMI_9 | SNV | M | 0.01307 |
| 10585703 | Rpp25 | chr9 | BMI_9 | eQTL | M | 0.00276 |
| 10599232 | Nkap | chrX | BMI_X | BOTH | C | 0.04527 |
| 10599321 | Zbtb33 | chrX | BMI_X | SNV | C | 0.01365 |
| 10599321 | Zbtb33 | chrX | BMI_X | SNV | M | 0.01049 |
| 10604199 | Cul4b | chrX | BMI_X | eQTL | C | 0.03413 |
| 10604199 | Cul4b | chrX | BMI_X | eQTL | M | 0.01667 |
| 10599498 | Utp14a | chrX | BMI_X | SNV | C | 0.02532 |
| 10599514 | Bcorl1 | chrX | BMI_X | SNV | C | 0.00396 |
| 10604922 | BC023829 | chrX | BMI_X | eQTL | C | 0.02768 |

| Probe ID | Gene Symbol | Male rho | Female Rho |
| --- | --- | --- | --- |
| 10364784 | Reep6 | 0.664 | 0.596 |
| 10367960 | Pex3 | 0.612 | 0.677 |
| 10378572 | Tlcd2 | 0.575 | 0.463 |
| 10393970 | Fasn | 0.671 | 0.447 |
| 10400941 | Dhrs7 | 0.682 | 0.559 |
| 10401181 | Rdh11 | 0.628 | 0.609 |
| 10429926 | Dgat1 | 0.678 | 0.654 |
| 10431124 | Pnpla5 | 0.701 | 0.548 |
| 10438262 | Slc25a1 | 0.523 | 0.385 |
| 10462630 | Pank1 | 0.638 | 0.470 |
| 10467162 | Pank1 | 0.667 | 0.655 |
| 10470751 | Slc27a4 | 0.696 | 0.607 |
| 10477090 | Tbc1d20 | 0.659 | 0.486 |
| 10486172 | Fam82a2 | 0.633 | 0.509 |
| 10487154 | Secisbp2l | 0.760 | 0.729 |
| 10488472 | 2310001A20Rik | 0.600 | 0.177 |
| 10506571 | Dhcr24 | 0.639 | 0.609 |
| 10514491 | Cyp2j12-ps | 0.576 | 0.640 |
| 10528207 | Cd36 | 0.602 | 0.557 |
| 10539894 | Mgll | 0.694 | 0.614 |
| 10540897 | Pparg | 0.669 | 0.658 |
| 10542857 | Far2 | 0.613 | 0.494 |
| 10557498 | Fam57b | 0.616 | 0.518 |
| 10558265 | Lhpp | 0.667 | 0.611 |
| 10569972 | Lass4 | 0.626 | 0.687 |
| 10578916 | Sc4mol | 0.643 | 0.362 |
| 10581824 | Fa2h | 0.755 | 0.591 |
| 10587988 | Gk5 | 0.646 | 0.559 |
| 10593776 | Nrg4 | 0.596 | 0.550 |
| 10600988 | Awat1 | 0.683 | 0.610 |

Additional file 2: Table S5: *Panx3* network gene correlation levels by sex

Additional file 2: Table S6: *Panx3* polymorphisms between Spret and FVB mice.

| Chromosome | Location | Panx3 exon | Nucleotide change | Amino acid change |
| --- | --- | --- | --- | --- |
| 9 | 37661708 | 4 | G545A | R182Q |
| 9 | 37664065 | 3 | G500C | S167T |
| 9 | 37668999 | 1 | C57G | D19E |

In all cases the first allele is the FVB allele, and the second allele is the SPRET allele.

Supplementary Figures

Additional file 2: Figure S1: 20 week papilloma burden by BMI for male and female mice.

Additional file 2: Figure S2: QTL effect for the strongest autosomal QTL for each phenotype by sex for raw and mean-centered phenotype values.

Additional file 2: Figure S3: Proximal and distal regions of chromosome 10 influence weight in opposing directions.

Additional file 2: Figure S4: Effect of sex-specific QTL on BMI.

Additional file 2: Figure S5: *Panx3* expression and tumor development.

Supplementary figure legends

Additional file 2: Figure S1: 20 week papilloma burden by BMI for male and female mice. BMI values and 20 week papilloma burdens are plotted for female (left) and male (right) mice. BMI values were mean-centered by sex. The relationship is significant for male mice and non-significant for female mice (p < 1e-7, rho = 0.38 for males; p > 0.05, rho = 0.09 for females).

Additional file 2: Figure S2: QTL effect for the strongest autosomal QTL for each phenotype by sex for raw and mean-centered phenotype values. Phenotype values (length, weight, and BMI) are plotted for the three most consequential autosomal QTL by genotype and sex. **A)** Raw and **B)** mean-centered by sex values are plotted for BMI_10, WEI_11, and LEN_11. Squares indicate the mean for that genotype, and the boundaries represent the 95% confidence interval around the mean.

Additional file 2: Figure S3: Proximal and distal regions of chromosome 10 influence weight in opposing directions. The effect of the proximal chromosome 10 weight QTL (WEI_10_PROX) and the distal QTL (WEI_10_DIST) are plotted in both sexes combined. Mean values by QTL genotype are depicted, as well as the 95% confidence interval around the mean. Weight values were mean centered for both sexes prior to analysis. The differences between genotypes are significant for both mean centered and raw values.

Additional file 2: Figure S4: Effect of sex-specific QTL on BMI. BMI mean and the 95% confidence interval are plotted for female, male, and combined mice by marker genotypes. BMI_3 is plotted in the upper panel, and BMI_9 in the lower. The differences between genotypes are significant for females for BMI_3, males for BMI_9, and the combined set for BMI_3.

Additional file 2: Figure S5: *Panx3* expression and tumor development. **A)** Pretreatment *Panx3* expression is correlated with papilloma burden at 20 weeks in male mice (p = 0.32, rho = 0.28) and is not associated in female mice (p > 0.05, rho = -0.09). **B)** Carcinoma-free survival in female mice by *Panx3* expression. *Panx3* expression groups were defined as the highest and lowest quartile of expression values. **C)** Carcinoma-free survival in male mice by *Panx3* expression. Grouping was assigned as in panel B.
